# Genetic architecture of two novel chicken breeds from Xinjiang: A whole-genome sequencing study on Ili gamecock and Yemili Chicken

**DOI:** 10.1016/j.psj.2026.106845

**Published:** 2026-03-21

**Authors:** Haichen Yang, Qianqian Liang, Peng Su, Göran Andersson, Erik Bongcam-Rudloff, Mahaba Rouzi, Lin Jiang, Jilong Han, Min Yang

**Affiliations:** a College of Animal Science and Technology, Shihezi University, Shihezi 832061, China; b National Germplasm Center of Domestic Animal Resources, Ministry of Science and Technology of the People's Republic of China, Institute of Animal Science, Chinese Academy of Agricultural Sciences (CAAS), Beijing 100193, China; c Department of Animal Biosciences, Swedish University of Agricultural Sciences, Uppsala 75007, Sweden; d Xinjiang Uygur Autonomous Regional Animal Husbandry Station, Urumqi 830000, China

**Keywords:** Indigenous chicken breeds, Genetic resources, Whole-genome sequencing, Selection signature analysis, Genetic diversity

## Abstract

Indigenous poultry genetic resources are crucial for breeding and food security. In Xinjiang, China, the Ili gamecock and Yemili chicken represent two indigenous breeds with distinct and valuable traits. The Ili gamecock is prized for its large body size and aggressive behavior, whereas the Yemili chicken shows remarkable adaptation to the cold environment of the Tacheng area, with strong disease resistance, and foraging ability suited to free-range grazing. As understanding their genetic basis is key to their conservation and sustainable use, we conducted whole-genome sequencing of 22 individuals from both breeds and integrated the data with 83 publicly available genomes to construct a comprehensive dataset of 12 global chicken populations. After identifying over 11.3 million high-quality SNPs, we assessed genetic diversity and population structure. Analyses revealed that the Ili gamecock is closely related to the Turpan gamecock, forming a distinct cluster. Selection signature analyses based on fixation index (FST) and nucleotide diversity ratio (π ratio) identified genomic regions under positive selection associated with aggressiveness and muscularity in gamecocks (e.g., *NELL1, SOX5, SEMA3A, KCNMA1*) and with stress response, intestinal integrity, and energy homeostasis in Yemili chickens (e.g.,*MAPK8IP3, HBEGF, PARD3, ATP6V1B2, ATP5PD*). This study provides a comprehensive genomic landscape of these two emerging Xinjiang breeds, elucidates their unique evolutionary histories, and offers valuable genetic resources for future conservation and breeding programs.

## Introduction

Chickens (*Gallus gallus domesticus*), among the earliest domesticated avian species (domesticated ∼10,000 years ago), have diversified into numerous breeds through prolonged artificial selection across varied geographical environments (Kijas et al., 2012; Xiang et al., 2014). China is a major repository of this diversity, hosting 116 recognized indigenous chicken breeds that constitute approximately 10% of the world’s chicken genetic resources (Millward, 2012). Among these, Xinjiang province harbors several valuable local breeds, including the Baicheng fatty chicken, Turpan gamecock and Niya black chicken. More recently, two distinctive indigenous genetic resources—the Ili gamecock and the Yemili chicken—have been identified in this region, exhibiting distinct phenotypic and adaptive traits. The domestic chicken remains one of the most economically significant agricultural animals worldwide, serving as a major source of protein and other by-products such as feathers. A cornerstone in avian genomics was laid in 2004 with the completion of the chicken genome sequencing project using the inbred line UCD001 and BAC-based sequencing technology by the International Chicken Genome Sequencing Consortium (Schmid et al., 2015). Subsequent efforts have yielded a high-quality reference genome of the red junglefowl (*Gallus gallus*), the wild ancestor of domestic chickens.

**Fig. 1:**
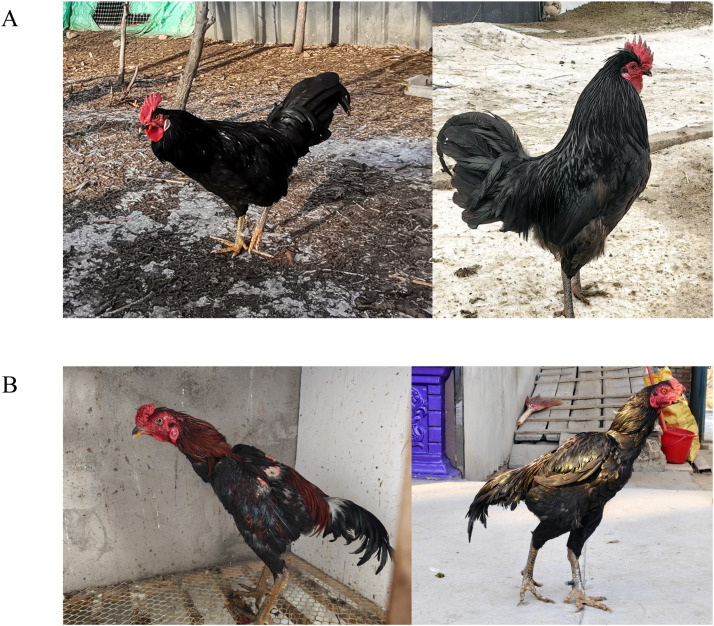
Appearance of the Yemili Chicken and Ili gameock. (A) Yemili chicken. (B) Ili gamecock.

The Ili gamecock, traditionally raised in the Ili Kazakh Autonomous Prefecture of northern Xinjiang, is characterized by its large and robust body size, well-developed pectoral and leg muscles, and pronounced aggressive behavior, holding significant cultural value among local Uyghur communities where cockfighting has been practiced for generations. This breed exhibits distinctive phenotypic traits including bean or “pimple” combs in males and flat silkworm-shaped combs in females, with underdeveloped tail feathers and tight body plumage. Due to its large body size, the breed also possesses potential meat production value (Fig. 1B). In parallel, the Yemili chicken represents an emerging genetic resource from Xinjiang, demonstrating remarkable adaptability to the unique natural environment of Emin County in Tacheng Prefecture—a region characterized by extremely hot summers and severely frigid winters (−30°C to 42°C), a climatic pattern distinctly different from other habitats of local chicken breeds in China. This breed exhibits a compact body, black plumage, short sturdy legs, and strong foraging ability suited to free-range grazing, with overall robustness (Fig. 1A). However, the genetic integrity of these native breeds is increasingly threatened by the introduction of commercial exotic lines, leading to genetic dilution and potential loss of valuable adaptive traits, such as disease resistance and environmental resilience. Therefore, the conservation and scientific study of these indigenous breeds are imperative not only for safeguarding invaluable genetic resources and cultural heritage but also for ensuring future agricultural sustainability and resilience.

To effectively conserve these threatened breeds and harness their unique traits, a thorough understanding of their genomic makeup is essential. Fortunately, recent genomic advances have enabled deeper insights into the genetic basis of local adaptation and diversity in chickens. For instance, For instance, comparative genomic approaches have identified novel genes related to thermal adaptation in chickens reared under different climatic conditions (Shi et al., 2023), while genome-wide SNP profiling has uncovered considerable genetic diversity in Nigerian indigenous chickens (Rachman et al., 2024). Nevertheless, comprehensive genomic information on the Ili gamecock and Yemili chicken remains scarce, limiting conservation and breeding efforts.

To address this gap, we conducted whole-genome sequencing of the Ili gamecock, Yemili chicken, and other Xinjiang breeds, integrating these data with publicly available genomes from diverse Chinese indigenous and commercial populations. Using population genetic and selection signature analyses, this study aimed to: 1) evaluate the genetic relationships among Xinjiang native chickens, other Chinese indigenous breeds, and commercial populations; 2)identify genetic variants and selection signatures associated with key traits in the Ili gamecock (e.g., aggression, muscularity) and Yemili chicken (e.g., cold adaptation, disease resistance); and (3) detect candidate genes underlying these economically and adaptively important phenotypes. Our findings are expected to elucidate the genetic mechanisms of adaptation in these unique breeds, contribute to their scientific conservation, and provide valuable genetic resources for future breeding programs aimed at enhancing resilience and productivity.

## Materials and methods

### Ethical statement

All animal experiments were authorized by the Biology Ethics Committee of Shihezi University. The ethic committee approval number is: A2020-34.

### Samples collection, DNA extraction, and SNP calling

Ili gamecock (YL) samples (*n* = 9) were collected from the farmers of Wutubulake Village, Dongmaili Town, Gongliu County, Xinjiang and Yemili chicken (YML) samples (*n* = 13) were obtained from the Emin County Hengxin Industrial Co., Ltd, Tacheng, Xinjiang. All individuals originated from the core conservation populations of their respective breeds. To ensure that the sampled individuals were representative of the purebred populations, for YL, only males displaying the typical breed characteristics were selected: large and robust body size, well-developed pectoral and leg muscles. For YML, individuals were chosen based on the breed-specific phenotype: compact body, black plumage, short and sturdy legs, strong foraging ability, and healthy and robust condition. DNA was extracted using the standard phenol-chloroform method (Kochl et al., 2005). Paired-end sequence data for all individuals were generated using the DNBSEQ T7 platform. Additionally, we collected whole-genome sequencing data of 83 individual chickens from public datasets. These breeds include Tibetan chicken (ZJ), Beijing you chicken (YOU), Turpan gamecock (TLF), Red jungle fowl (RJF), Rhode island red (RIR), Niya chicken (NY), Huaixiang chicken (HX), and Changting hetian chicken (CT) (Supplementary Table S1).

The reference genome used was Gallus_gallus.GRCg6a (Gallus gallus genome assembly GRCg6a - NCBI - NLM,GCF_000002315.5). After quality filtering of the original FASTQ files, whole-genome sequences were aligned using BWA v1.0.3 to generate Binary Alignment Map (.bam) files. Subsequently, GATK v4.2.0 was used to construct gVCF files for each sample, and gVCF files were merged using GLnexus v1.4.1 (Babadi et al., 2023). Finally, we removed SNP sequencing and alignment errors using the Bcftools v1.13 view module with filters: "QD < 2.0, QUAL < 30.0, SOR > 3.0, FS > 60.0, MQ < 40.0, MQRankSum < −12.5, and ReadPosRankSum < −8.0″. Variants with a call rate > 80% and minor allele frequency < 0.05 were further filtered out using bcftools for subsequent analyses.

### Genetic diversity and population structure

Using PLINK v1.90 (Purcell et al., 2007), we calculated the observed heterozygosity (Ho), expected heterozygosity (He), and identified the regions of homozygosity and inbreeding coefficient (FROH). Used the “–hardy” parameter in PLINK v1.90 to calculate Ho and He, and the “–maf” parameter to calculate minor allele frequencies (MAF). Linkage disequilibrium (LD) decay was estimated using Haploview v4.2 (Barrett, 2009). The squared correlation coefficient (r²) between pairwise SNPs was calculated with the parameter "-n -dprime -minMAF 0.05″. ROH analysis parameters: Minimum number of SNPs (–homozyg-snp 50), indicating the minimum number of SNPs required within a ROH segment; Maximum allowed gap between contiguous homozygous segments (–homozyg-gap 1000); Minimum ROH length (–homozyg-kb 100); Maximum allowed heterozygous SNPs within contiguous homozygous segments (–homozyg-window-het 5); ROH sliding window size (–homozyg-window-snp 50); Maximum number of missing SNPs allowed within a sliding window (–homozyg-window-missing 5); and Confidence threshold for determining ROHs (–homozyg-window-threshold 0.05). Principal component analysis was performed on the dataset after filtering out SNPs with high linkage disequilibrium (r² ≥ 0.1) using PLINK v1.90 with the parameter "–indep-pairwise 50 10 0.1″. Using VCF2Dis v1.54 (Xu et al., 2025), we calculated the p-distance matrix. Based on this matrix, we constructed a neighbor-joining (NJ) phylogenetic tree using ATGC: FastME. We visualized the NJ-tree using the iTOL web server (Letunic and Bork, 2016). Ancestry composition analysis was performed using the ADMIXTURE software (Patterson et al., 2012), where population stratification can be calculated based on the proportion of genome-wide genetic variation and variation from each individual in each of K ancestors.

### TreeMix analysis

The TreeMix software uses the maximum likelihood method to infer genetic relationships between populations, while accounting for potential gene flow (Das et al., 2020). The number of gene flow events (m) was preset between 1 and 5, with each value replicated 5 times. RJF was utilized as the root of the phylogenetic tree in the TreeMix analysis. To identify the optimal m, the TreeMix results were evaluated using the R package OptM. The largest ΔM, corresponding to *m* = 3, was selected as the optimal m and plotted using plotting funcs.R in TreeMix.

### Selective sweep analysis

This study conducted FST analysis on SNP data from all chicken populations in this study, including YML, commercial chickens, YL, and other breeds, with selection signals identified using allele frequencies at variable sites in 50 kb genomic windows and a 5 kb step size. Two sets of environment and trait-specific comparative groups were established: select groups of gamecocks and non-gamecocks, as well as groups of YML and non-YML. To explore candidate genomic regions and genes associated with gamecock-specific traits, we compared gamecock populations (YL and TLF) with non-gamecock conspecifics to clarify the genetic mechanisms underlying gamecock characteristics (aggressiveness and muscularity). Concurrently, to investigate the genetic basis of Yemili chickens (YML) adaptation to the dual extreme natural environment of Emin County (42°Cultra-high temperature and −30°Cultra-low temperature) (https://www.tianqi24.com/emin/history.html), we performed parallel selective sweep analysis comparing Yemili chickens with populations adapted to a single extreme environment (the other 11 chicken breeds in this study). These contrast populations only adapt to a single type of extreme environment (e.g., ZJ to ultra-cold only, WC to ultra-hot only) or mild/stable environments, and have no evolutionary experience with the dual extreme environmental stress of Emin County. FST analysis was conducted using VCFtools v0.1.13 (Danecek et al., 2011; Shen et al., 2020). In each comparison, the top 1% genomic regions with the highest scores are considered potential selection regions.The FST calculation formula is as follows: where MSG is the mean square error within groups, MSP is the mean square error between groups, and nc is the corrected average sample size for the entire population. This method is primarily applicable for detecting signals of selection between different populations.

$$\text{FST}=\frac{\text{MSP}-\text{MSG}}{\text{MSP}+(n_{c}-1)\text{MSG}}$$

Nucleotide diversity (Pi) analysis is an essential measure of population diversity. Pi was calculated separately for the YL populations, the Pi ratio of the selected population and the compared population was calculated as the π ratio. Finally, the 1% high-ranking values were taken in ascending order to intersect with the FST results, where S denotes the number of segregating loci and hj denotes the heterozygosity of the j segregating locus.

$$\text{Pi}=\sum_{j=i}^{S}h_{j}$$

$$\pi \;\text{ratio}=\frac{\text{Pi}(\text{Gamecocks})}{\text{Pi}(\text{Non}-\text{gamecocks})}$$

### Enrichment analysis for candidate genes

The intersection of regions identified by both πratio and FST analyses was determined to pinpoint robust selection signatures. Then subsequently annotated these results using the chicken (GRCg6a, GCA_000002315.5) genome. Then, we use KOBAS v3.0 (http://bioinfo.org/kobas) to perform gene ontology (GO) enrichment analysis and Kyoto Encyclopedia of Genes and Genomes (KEGG) pathway analysis on the intersecting genes (Bu et al., 2021). Among them, GO enrichment analysis is used to predict and elucidate the roles of gene products in molecular functions, biological processes, and cellular components (Ashburner et al., 2000). KEGG pathway enrichment analysis is used to identify the main biochemical and signaling pathways that genes are involved in (Kanehisa et al., 2016). Adjust P value (method) to less than 0.05.

## Results

### Variation detection and genetic diversity

In this study, we conducted whole-genome sequencing of 105 chickens and identified a total of 13,356,328 SNPs, of which 1,800,544 remained after pruning. Genetic diversity analysis showed that, for chicken breeds in the Xinjiang region, Ho ranged from 0.1693 to 0.3533, whereas genomic FROH ranged from 0.0029 to 0.0628 (Table 1). In contrast, Chinese native chickens exhibited lower Ho (0.1133–0.1757). The Ho of the RJF was 0.0983, which was lower than its He. Comparative analyses indicate that YML and the YL maintain high levels of genetic diversity relative to chickens from other regions (Table 1, Supplementary Table S3).

**Table 1:** Location information and genetic diversity of twelve chicken breeds.

Name	Breed	Collection region	Number	Observed heterozygosity (Ho)	Expected heterozygosity (He)	Inbreeding coefficient(FROH)
Tibetan chicken	ZJ	Tibetan (China)	6	0.1133	0.0818	0.0284
Ili gamecock	YL	Xinjiang (China)	9	0.3533	0.2207	0.0029
Yemili chicken	YML	Xinjiang (China)	13	0.2767	0.2114	0.0474
Turpan gamecock	TLF	Xinjiang (China)	6	0.1841	0.1653	0.0628
Niya chicken	NY	Xinjiang (China)	5	0.1693	0.1558	0.0539
Red jungle fowl	RJF	China&Indonesia&India	15	0.0983	0.1722	0.1594
Beijing you chicken	YOU	Beijing (China)	6	0.1434	0.1439	0.1682
Huaixiang chicken	HX	Guangdong (China)	10	0.1607	0.1635	0.0369
Changting hetian chicken	CT	Fujian (China)	6	0.1757	0.1656	0.0583
Wenchang chicken	WC	Hainan (China)	10	0.1656	0.1612	0.0356
Rhode island reds chicken	RIR	China	10	0.1161	0.1071	0.3283
White leghorn	WLL	China	10	0.0737	0.0951	0.4416

**Fig. 2:**
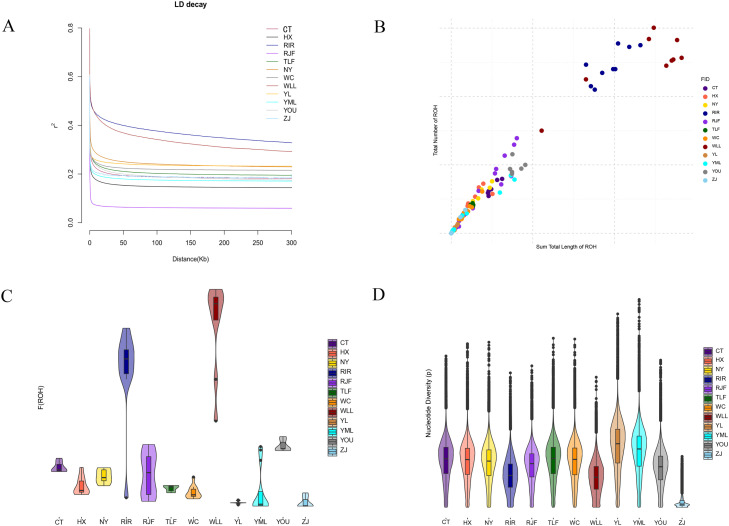
Genetic diversity among different chicken populations. (A) Genome-wide LD decay patterns across all studied populations. (B) Total number of ROH segments detected in each population. (C) Box plots of the FROH for each group. (D) Nucleotide diversity Pi for each group.

To characterize LD patterns across populations and infer the strength of selection, we evaluated LD decay (Fig. 2A). RJF exhibited the fastest decay rate, local breeds typically decayed faster, while commercial chickens exhibited a slower decay rate. This suggests that the two commercial chicken populations experienced relatively strong selection pressure. ROH provide an indicator of the degree of inbreeding in animals. Longer ROH segments indicate more recent inbreeding events, whereas shorter segments reflect more ancient inbreeding. We analyzed ROH patterns and genomic FROH in 12 chicken breeds (Fig. 2B, C). ROH were widely distributed across all populations, with significant differences in the number and length of ROH and in FROH among breeds. The WLL population showed the longest ROH and the highest number of ROH segments, as well as the highest FROH, followed by RIR. In contrast, YL had the shortest ROH segments, the fewest ROH, and the lowest genomic FROH. The Pi shows that the YL has a high Pi, second only to YML (Fig. 2D).

### Population structure analysis

**Fig. 3:**
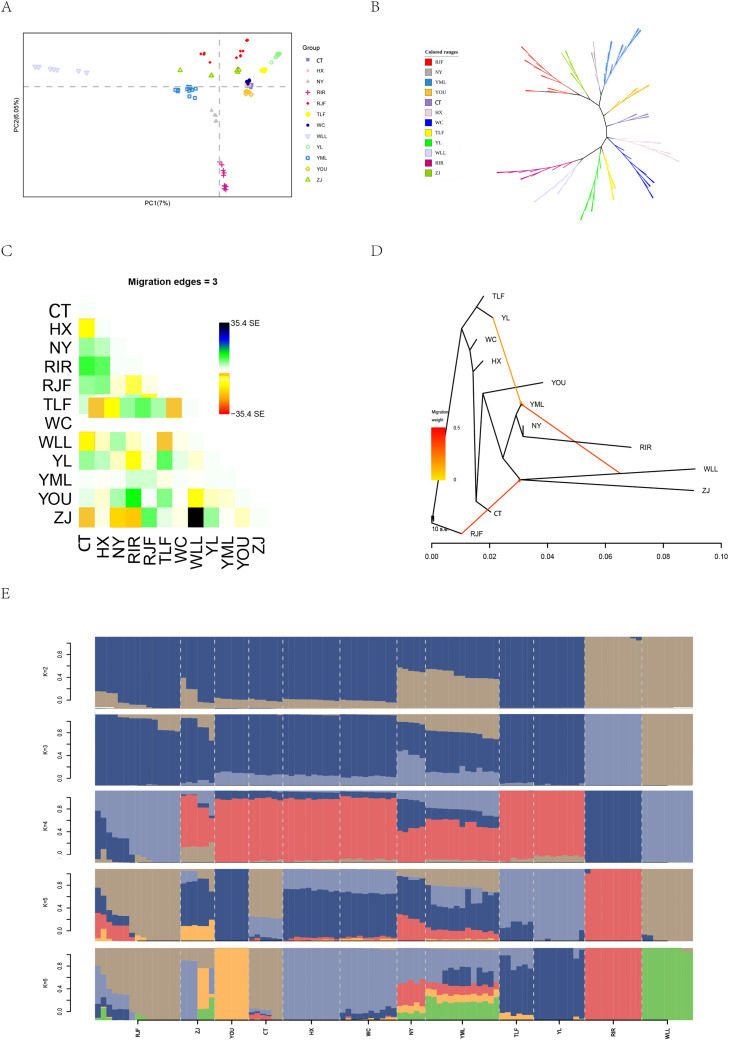
Population genetic analyses of 12 chicken breeds. (A) Principal component analysis. (B) Neighbor-joining tree. (C-D) Treemix for each group. *n* = 3. (E) Genetic structure of chicken breeds. The length of each colored segment represents the proport.

We performed PCA analysis on SNP data of 12 chicken flocks (Fig. 3A). The first two principal components (PC1 and PC2) explained 7% and 6.05% of the total variance, respectively. The PCA results showed that overlaps among WC (Hainan), CT (Fujian) and HX (Guangdong) populations, which may be attributed to their geographic proximity in southeastern China. In contrast, other populations showed clear separation. Notably, YL was positioned close to TLF in the PCA plot. We constructed an NJ tree based on the same dataset (Fig. 3B). The results were basically consistent with PCA results, YL was adjacent to TLF, while YML was adjacent to NY. According to the OptM results (Figure S2, Supplementary Figures), the optimal number of admixture events in TreeMix was three, which was the gene flow from RJF to other populations (WLL), WLL to YML and YL to YML (Fig. 3C, D). Considering the evolution process among varieties, the population structure of different populations was further analyzed by using hybrid software (Fig. 3E). At *K* = 2, only commercial chickens could be clearly distinguished. At *K* = 3, the cross validation (CV) value is the lowest (Figure S1, Supplementary Figures). YML and NY, as well as YL and TLF, shared similar ancestral components. Notably, YL and TLF exhibited more homogeneous genetic backgrounds with minimal influence from other breeds. At *K* = 6, only HX, RIR, WLL and YOU have a single ancestral component, followed by YL individuals. In contrast, extensive population admixture was detected in the remaining groups.

### Population selection s**ignatures** and adaptive evolution analysis

For both comparisons, FST and π ratio were calculated in 50 kb windows with a 5 kb step size; the top 1% of FST values and top 1% of |log2(π ratio)| values were considered potential selection regions.

**Fig. 4:**
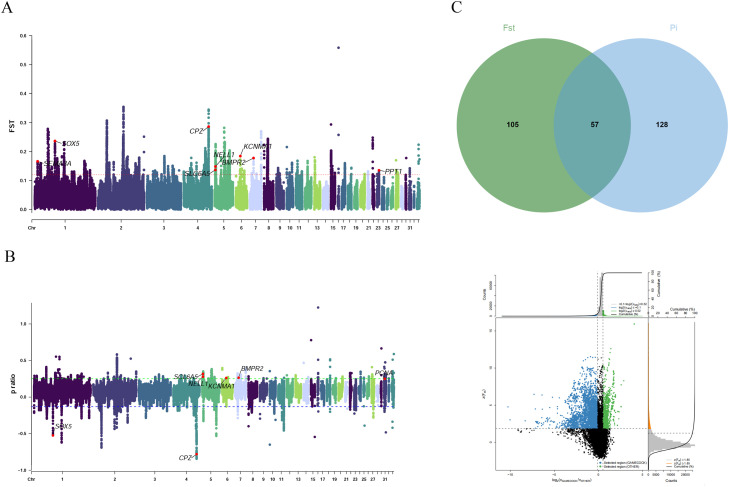
Genomic selection signatures and adaptive evolution analysis (GAMECOCK) : (A) Manhattan diagram of FST. (B) Manhattan diagram of π ratio. (C) Number of genes jointly screened Distribution of dot plot of up and FST values (top 1% outliers). (D) Distribution of dot plot of π ratio and FST values (top 1% outliers), Chickens were categorized into two groups: gamecocks (GAMECOCK) and all other non-gamecock breeds (Others).

For gamecocks, the top 1% of FST values identified 162 candidate genes (Supplementary Table S4), and the top 1% of |log2(π ratio)| values identified 185 candidate genes (Supplementary Table S5). The intersection of these two gene sets yielded 57 common genes, including *CPZ, SOX5, NELL1, BMPR2, SLC6A5, SEMA3A, PPT1* and *KCNMA1* (Fig. 4A-D, Supplementary Table S6).

**Fig. 5:**
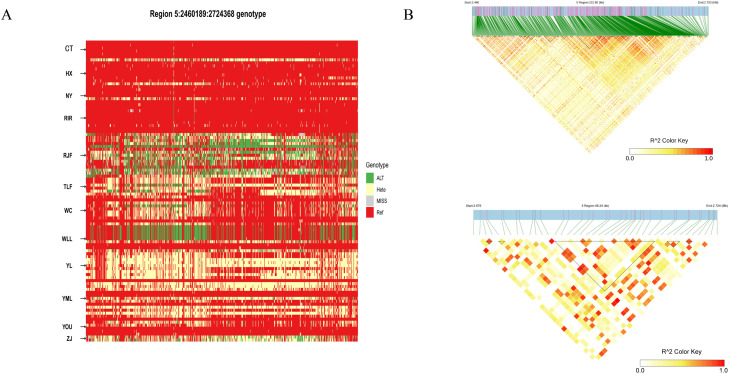
Haplotype Analysis: (A) Heatmap shows the degree of LD in the region of *NELL1* gene. (B) Genotype Heatmap of the *NELL1* gene. ALT denotes a homozygous mutation, HTET denotes a heterozygous mutation, REF denotes no mutation, and MISS denotes a deletion.

Subsequently, we examined the expression levels of the *NELL1* gene across various chicken tissues using the ChickenGTEx Atlas (https://ngdc.cncb.ac.cn/chickengtex/home). We observed high expression of *NELL1* in the brain, prompting us to conduct generate a haplotype heatmap and linkage disequilibrium (LD) analysis for the *NELL1* gene (Figure S3, Supplementary Figures). To further characterize genetic variation in key genes for aggression and muscular traits, we constructed a haplotype heatmap for the *NELL1*. The results showed that specific haplotypes of *NELL1* were significantly enriched in gamecocks compared to non-gamecocks (Fig. 5A), indicating strong selective pressure on these haplotypes during the domestication and breeding of Ili gamecocks.Similarly, we identified numerous linkage regions in the *NELLl* linkage disequion analysis and discovered linkage regions in areas with the highest FST scores in previous analyses. These regions may be functionally associated with influencing aggressive behavior in gamecocks.

**Fig. 6:**
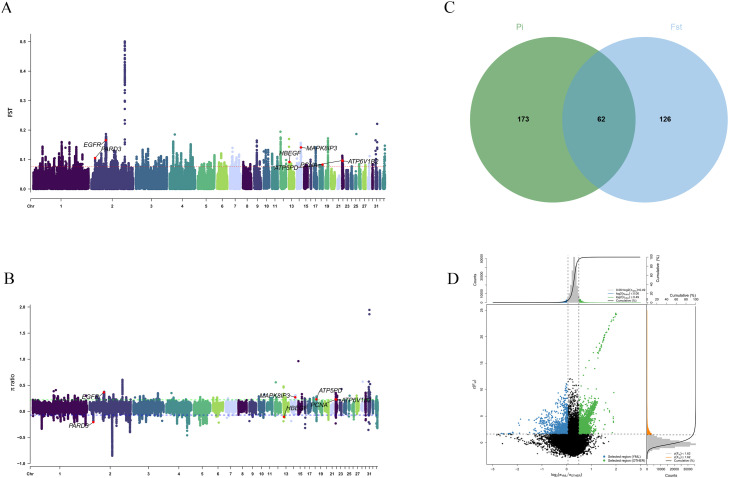
Genomic selection signatures and adaptive evolution analysis (YML): (A) Manhattan diagram of FST and π ratio. (B) Number of genes jointly screened. (C) Distribution of dot plot of π ratio and FST values (top 1% outliers). Chickens were categorized into two groups: Yemili chicken (YML) and all other non-Yemili breeds (Others).

For YML, the top 1% of FST values identified 235 candidate genes, and the top 1% of |log2(π ratio)| values identified 188 candidate genes (Supplementary Table S7-8). The intersection of these two gene sets yielded 62 common genes, including *MAPK8IP3, HBEGF, PARD3, ATP6V1B2*, and *ATP5PD* (Fig. 6A-D, Supplementary Table S9).

**Fig. 7:**
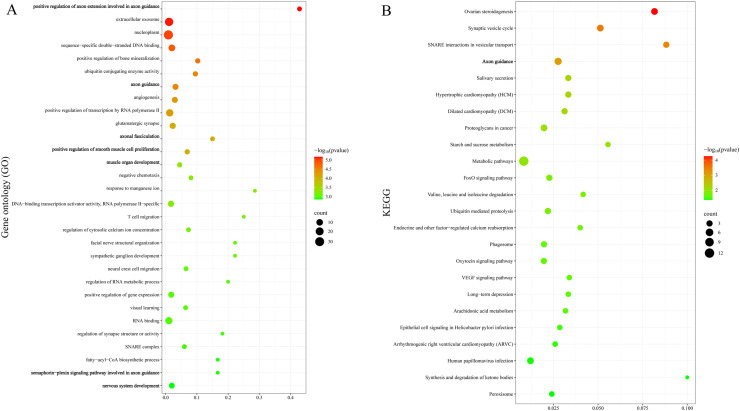
Genes selected for gamecock enrichment analysis: (A) Kyoto encyclopedia of genes and genomes. (B) Gene Ontology.

We further performed Gene Ontology (GO) enrichment analysis and Kyoto Encyclopedia of Genes and Genomes (KEGG) pathway analysis on these candidate genes using KOBAS v3.0. For gamecocks, genes and pathways related to neurodevelopment and growth were significantly enriched, such as "positive regulation of axon extension involved in axon guidance" and the "Axon guidance" pathway (Fig. 7A-B) (Supplementary Table S10). For YML, pathways related to self-adaptation and immunity were enriched, such as༂MAPK signaling pathway༂and the "Tight junction" pathway (Supplementary Table S11, Figure S4, Supplementary Figures).

## Discussion

The conservation and utilization of indigenous poultry genetic resources are paramount for enhancing genetic diversity, fostering climate resilience, and ensuring sustainable global food security. This study conducts a comprehensive genomic analysis of Xinjiang local chicken breeds, with a particular focus on the YL and the YML. By integrating assessments of genetic diversity, population structure, and selection signals, we reveal their unique genetic characteristics and adaptive evolutionary mechanisms, providing critical genomic insights for the conservation and utilization of these important genetic resources.

### Genetic diversity and demographic history of Xinjiang chickens

Our analyses revealed that the YL and YML maintain notably high levels of genomic diversity. Their Ho and Pi were among the highest across all studied populations, while their FROH and ROH were the lowest. This genetic profile suggests a historically large effective population size and limited recent inbreeding, which has preserved a broad genetic base likely contributing to their adaptability (Liu et al., 2021). In contrast, commercial breeds like WLL exhibited the longest ROH segments and highest inbreeding coefficients, a clear genomic signature of intense artificial selection and population bottlenecks associated with modern breeding for production traits. The rapid LD decay observed in RJF and local breeds, compared to the slower decay in commercial lines, further corroborates a history of weaker directional selection in the indigenous populations, allowing for the maintenance of ancestral genetic variation (Islam et al., 2019; Bendesky et al., 2024). Meanwhile, it is important to acknowledge that the sample sizes for some breeds in this study were relatively small. This constitutes a limitation of the current study. Genetic diversity metrics, such as Pi and Ho, as well as inbreeding coefficients like FROH, are sensitive to sample size. Small sample sizes can lead to high sampling variance and may not fully represent the allelic diversity present within a breed. Consequently, while the genetic parameters reported here offer a preliminary glimpse into the genomic characteristics of these breeds, they should be interpreted with appropriate caution. Increasing the sample size in future investigations would enable a more comprehensive and robust assessment of their genetic makeup.

Population structure analyses (PCA, NJ tree, ADMIXTURE) delineated clear genetic boundaries and historical relationships. The close genetic affinity between YL and TLF indicates a shared ancestry, forming a distinct Xinjiang gamecock lineage. This finding is consistent with previous research suggesting a specific genetic cluster for gamecocks in this region (Ren et al., 2023). The genetic similarity between YML and NY, despite geographical separation, points to a common origin with subsequent divergence, a pattern also observed in other black-feathered chicken resources in China (Gao et al., 2017; Yang et al., 2025). TreeMix analysis revealed significant historical gene flow events, including from RJF to commercial lines and from YL to YML. Importantly, we also detected signals of modern gene flow from commercial WLL into the YML, highlighting a tangible contemporary threat of genetic dilution to indigenous genomic integrity and underscoring the urgency of targeted conservation strategies.

### Genetic basis of key traits in gamecocks

The YL is characterized by two core breeding traits: a robust, muscular physique and pronounced aggressive behavior. Our selective sweep analysis identified a suite of 57 candidate genes under selection in gamecocks, functionally converging on skeletal-muscular development and neural regulation pathways. This suggests an integrated genetic architecture for this complex phenotype, where selection has acted on a coordinated network rather than isolated traits.

Key genes such as *CPZ, SOX5, BMPR2, MSX1, IGF1*, and *NELL1* are central regulators of osteogenesis, chondrogenesis, and myogenesis (Xue et al., 2017; Xu et al., 2018; Luo et al., 2020; Lyu et al., 2023; Xu et al., 2025), providing a direct genetic basis for the gamecocks' formidable physique. Among these, the *NELL1* gene stood out due to its exceptionally high linkage disequilibrium and enriched haplotypes specifically in gamecock populations. *NELL1* is known to promote osteogenic differentiation while inhibiting adipogenesis (Wang et al., 2017; Yin et al., 2023), and acts synergistically with the *BMPR2*-mediated BMP signaling pathway to enhance skeletal and muscular development (Mao et al., 2022; Yin et al., 2023). Crucially, both *NELL1* and *BMPR2* have also been implicated in neural synapse development (Kashima et al., 2016; Kim et al., 2020). This dual role suggests a potential genetic coupling mechanism: selection for combat-related physicality may have co-selected for underlying neurological pathways that predispose to aggression, representing a compelling case of integrated evolutionary adaptation.

Concurrently, several other candidate genes are primarily associated with the neural substrate of aggressive behavior. *SEMA3A* guides axonal pathfinding and synaptic plasticity (Tran et al., 2007; Yang et al., 2024), and its dysregulation can affect neural circuits governing social behavior (Du et al., 2022). *PPT1* and *SLC17A6* (*VGLUT2*) are critical for synaptic function and glutamatergic neurotransmission (Sarkar et al., 2020; Su et al., 2024), while *KCNMA1* regulates neuronal excitability and is linked to impulsivity (Heukelum et al., 2021; Dinsdale et al., 2023). The significant enrichment of the "Axon guidance" underscores the importance of precise neural wiring. Therefore, the gamecock phenotype likely emerged from coordinated selection on a genetic network that synchronously enhanced physical capacity (via developmental genes like *NELL1* and *SOX5*) and aggressive propensity (via neural genes like *SEMA3A* and *KCNMA1*).

### The environmental adaptation of Yemili chicken

Surviving the harsh, cold winters of the Tacheng region necessitates robust physiological adaptations. Our selective sweep analysis in YML identified 62 candidate genes, with significant enrichment in pathways central to environmental resilience, notably the MAPK signaling pathway and tight junction formation.

This study has identified multiple pathways and genes significantly enriched in association with cold resistance and reproduction, such as *MAPK8IP3, HBEGF, EGFR*, and *PCNA*. Temperature profoundly influences animal growth, development, and reproduction. Generally, cold stress limits reproductive nutrition by increasing energy expenditure for thermoregulation, while disrupting ovarian function and immune responses. This not only reduces egg production and quality in laying hens but also adversely affects the birds' overall vitality (Mohebbifar et al., 2025).

Among these, the MAPK signalling pathway plays a central role in responding to cold stress. Cold temperatures not only affect immune function and intestinal barrier protection via the MAPK pathway but also significantly regulate the reproductive axis, a function widely demonstrated in the environmental adaptation of high-altitude species (Qiao et al., 2023; Li et al., 2025a). In broilers, intermittent mild cold stimulation enhances immune responses by activating the MAPK pathway while regulating the expression of cold-induced proteins (Xing et al., 2024). Furthermore, 72-hour cold stress significantly upregulates gene expression of tight junction proteins (e.g., Occludin, zonula occludin 1) in the jejunum, suggesting MAPK may indirectly maintain intestinal barrier function by regulating related pathways (Zhou et al., 2021). These findings align with subsequent observations of reciprocal regulation between reproductive function and intestinal immunity. Notably within the MAPK pathway, the key interacting protein *MAPK8IP3* is recognised for mediating cellular adaptation to stressors like cold temperatures via regulatory networks involving non-coding RNAs (Wan et al., 2022), thereby substantiating the MAPK pathway's role in cold environments. The gene *HBEGF*, also implicated in cold response, functions within the estrogen signaling pathway. By promoting estrogen synthesis via the EGFR-cAMP-PKA axis, *HBEGF* likely supports sustained ovarian function and egg production under conditions where cold stress typically suppresses reproduction. This dual role of the MAPK pathway exemplifies an integrated adaptation that balances survival and fertility (Huang et al., 2022).

The intestinal epithelium represents a critical frontline defense, particularly vulnerable under extreme cold (Gilani et al., 2021). Our findings highlight the importance of tight junction maintenance for barrier integrity. Genes such as *PARD3* are essential for the proper assembly of tight junction complexes (Liu et al., 2023), while *PCNA* is involved in epithelial cell proliferation and repair. Dysregulation of these genes, as observed under cold stress, leads to the downregulation of junctional proteins (e.g., occludin, ZO-1) and can induce epithelial-mesenchymal transition, thereby increasing intestinal permeability and systemic infection risk (Peng et al., 2022; Vohra et al., 2025). The preservation of this barrier is thus a key adaptive trait for maintaining overall health in harsh climates. Collectively, these mechanisms constitute the primary defence line in the gut of the Leghorn chicken against pathogen invasion and maintenance of intestinal health. Effective immune defense is energetically costly, especially under the metabolic strain of thermoregulation. The Yemili chicken exhibits genetic adaptations that optimize energy allocation for immunity. Effective phagosome activation constitutes a vital immune mechanism for eliminating invading pathogens (Shi et al., 2023). Genes such as *ATP5PD*, involved in oxidative phosphorylation, ensure efficient ATP production to fuel immune cell activity (Mai et al., 2021; Cao et al., 2025). *ATP6V1B2*, a subunit of the V-ATPase, contributes to lysosomal acidification—a process vital for phagosomal pathogen degradation. Furthermore, *TPK1* links energy metabolism with immune regulation through pathways like mTOR signaling and metabolic reprogramming (Jessulat et al., 2021). This integration ensures that immune surveillance and effector functions of Yemili chicken remain robust even under energetically constrained conditions.

In summary, the adaptation of the Yemili chicken constitutes a network of synergistic multi-level mechanisms: on one hand, it enhances cold tolerance by activating stress signalling pathways such as MAPK; on the other, it maintains intestinal barrier integrity and prevents immune dysregulation by reinforcing the structure and function of tight junctions; simultaneously, under energy-restricted conditions, it ensures stable immune defence through efficient phagocytosis and cellular metabolic reprogramming. These insights offer valuable genetic targets for breeding programs aimed at enhancing stress tolerance and productivity in poultry.

## Conclusions

This study uncovers the genetic basis of phenotypic evolution in YL Gamecock (combat-driven selection) and ecological adaptation in YML Chicken (stress-metabolism balance). These findings support *in site* conservation and offer precise gene targets (e.g., *NELL1, MAPK8IP3*) for breeding climate-resilient, productive poultry.

## Fundings

This research was funded by the Shihezi University International Science and Technology Cooperation Project (No. GJHZ202307), 2025 Thematic Case of China Academic Degrees and Graduate Education Development Center (CDGDC) (ZT-2510759002), the Open Project of the State Key Laboratory of Animal Biotech Breeding (No. 2024SKLAB 6-107), and Breeding Special Project of Shihezi University (YZZX202005).

## CRediT authorship contribution statement

**Haichen Yang:** Writing – original draft, Visualization, Formal analysis, Data curation. **Qianqian Liang:** Writing – original draft, Visualization, Validation, Software, Methodology. **Peng Su:** Writing – review & editing, Software. **Göran Andersson:** Writing – review & editing, Data curation. **Erik Bongcam-Rudloff:** Writing – review & editing, Data curation. **Mahaba Rouzi:** Visualization, Resources, Methodology, Conceptualization. **Lin Jiang:** Writing – review & editing, Supervision. **Jilong Han:** Writing – review & editing, Validation, Resources, Project administration, Conceptualization. **Min Yang:** Writing – review & editing, Writing – original draft, Validation, Supervision, Resources, Project administration, Conceptualization.

## Disclosures

The authors declare that they have no known competing financial interests or personal relationships that could have appeared to influence the work reported in this paper.

## Appendix Supplementary materials

Image, application 1

Image, application 2

## Footnotes

Supplementary material associated with this article can be found, in the online version, at doi:10.1016/j.psj.2026.106845.

## References

[bib0001] Ashburner M., Ball C.A., Blake J.A., Botstein D., Butler H., Cherry J.M., Davis A.P., Dolinski K., Dwight S.S., Eppig J.T., Harris M.A., Hill D.P., Issel-Tarver L., Kasarskis A., Lewis S., Matese J.C., Richardson J.E., Ringwald M., Rubin G.M., Sherlock G. (2000). Gene ontology: tool for the unification of biology. Nat. Genet..

[bib56] Babadi M., Fu J.M., Lee S.K. (2023). GATK-gCNV enables the discovery of rare copy number variants from exome sequencing data. Nat. Genet..

[bib0002] Barrett J.C. (2009). Haploview: visualization and analysis of SNP genotype data. Cold Spring Harb. Protoc..

[bib0003] Bendesky A., Brew J., Francis K.X. (2024). The main genetic locus associated with the evolution of gamecocks is centered on ISPD. G3 (Bethesda).

[bib0004] Bu D., Luo H., Huo P., Wang Z., Zhang S., He Z., Wu Y., Zhao L., Liu J., Guo J., Fang S., Cao W., Yi L., Zhao Y., Kong L. (2021). KOBAS-i: intelligent prioritization and exploratory visualization of biological functions for gene enrichment analysis. Nucleic Acids Res..

[bib0005] Cao F., Liu B., Hou X., Shi J., Li P., Zhang X., Zhang S., Zhao Q. (2025). Maternal polystyrene nanoplastics suppress zebrafish offspring development and locomotion through mitochondrial dysfunction. Environ. Pollut..

[bib0006] Danecek P., Auton A., Abecasis G., Albers C.A., Banks E., DePristo M.A., Handsaker R.E., Lunter G., Marth G.T., Sherry S.T., McVean G., Durbin R. (2011). The variant call format and VCFtools. Bioinformatics.

[bib0007] Das R., Ivanisenko V.A., Anashkina A.A., Upadhyai P. (2020). The story of the lost twins: decoding the genetic identities of the Kumhar and Kurcha populations from the Indian subcontinent. BMC Genet..

[bib0008] Dinsdale R.L., Roache C.E., Meredith A.L. (2023). Disease-associated KCNMA1 variants decrease circadian clock robustness in channelopathy mouse models. J Gen Physiol.

[bib0009] Du Y., Shi Y., Wang X., Song H., Wang X., Hao Y., Zhao Y., Guo X., Shi M., Gong M., Song L., Wang S., Gao Y., Shi H. (2022). Hippocampal semaphorin 3B improves depression-like behaviours in mice by upregulating synaptic plasticity and inhibiting neuronal apoptosis. J. Neurochem..

[bib0010] Gao Y., Jia X., Tang X., Fan Y., Lu J., Huang S., Tang M. (2017). The genetic diversity of chicken breeds from Jiangxi, assessed with BCDO2 and the complete mitochondrial DNA d-loop region. PLoS One.

[bib0011] Gilani S., Chrystal P.V., Barekatain R. (2021). Current experimental models, assessment and dietary modulations of intestinal permeability in broiler chickens. Anim. Nutr..

[bib0012] Heukelum S.V., Geers F.E., Tulva K., van Dulm S., Beckmann C.F., Buitelaar J.K., Glennon J.C., Vogt B.A., Havenith M.N. (2021). Structural Degradation in Midcingulate Cortex Is Associated with Pathological Aggression in Mice. Brain Sci..

[bib0013] Huang J., Duan C., Jin S., Sheng C., Wang Y., Yue Z., Guo B. (2022). HB-EGF induces mitochondrial dysfunction via estrogen hypersecretion in granulosa cells dependent on cAMP-PKA-JNK/ERK-Ca(2+)-FOXO1 pathway. Int. J. Biol. Sci..

[bib0014] Islam M.A., Osman S.A.M., Nishibori M. (2019). Genetic diversity of Bangladeshi native chickens based on complete sequence of mitochondrial DNA d-loop region. Br. Poult. Sci..

[bib0015] Jessulat M., Amin S., Hooshyar M., Malty R., Moutaoufik M.T., Zilocchi M., Istace Z., Phanse S., Aoki H., Omidi K., Burnside D., Samanfar B., Aly K.A., Golshani A., Babu M. (2021). The conserved Tpk1 regulates non-homologous end joining double-strand break repair by phosphorylation of Nej1, a homolog of the human XLF. Nucleic Acids Res..

[bib0016] Kanehisa M., Sato Y., Kawashima M., Furumichi M., Tanabe M. (2016). KEGG as a reference resource for gene and protein annotation. Nucleic Acids Res..

[bib0017] Kashima R., Roy S., Ascano M., Martinez-Cerdeno V., Ariza-Torres J., Kim S., Louie J., Lu Y., Leyton P., Bloch K.D., Kornberg T.B., Hagerman P.J., Hagerman R., Lagna G., Hata A. (2016). Augmented noncanonical BMP type II receptor signaling mediates the synaptic abnormality of fragile X syndrome. Sci. Signal.

[bib0018] Kijas J.W., Lenstra J..A., Hayes B., Boitard S., Porto Neto L.R., San Cristobal M., Servin B., McCulloch R., Whan V., Gietzen K., Paiva S., Barendse W., Ciani E., Raadsma H., McEwan J., Dalrymple B. (2012). Genome-wide analysis of the world's sheep breeds reveals high levels of historic mixture and strong recent selection. PLoS Biol..

[bib0019] Kim H.R., Kim D..H., An J.Y., Kang D., Park J.W., Hwang E.M., Seo E.J., Jang I.H., Ha C.M., Lee B.J. (2020). NELL2 Function in axon development of hippocampal neurons. Mol. Cells.

[bib0020] Kochl S., Niederstatter H., Parson W. (2005). DNA extraction and quantitation of forensic samples using the phenol-chloroform method and real-time PCR. Methods Mol. Biol..

[bib0021] Letunic, I., & Bork, P. (2016). Interactive tree of life (iTOL) v3: an online tool for the display and annotation of phylogenetic and other trees. *Nucleic acids research, 44*(W1), W242–W245. 10.1093/nar/gkw290 PMC4987883 27095192

[bib0022] Li C.Y., Meng X.T., Liu Z.P., Zhang X., Zhou B., Zhao H.Y., Zhao J.D., Fu G.W., Chang Y.C., Gong S.R., Huo J.L., Zhao G.Y. (2025). Integrative transcriptomic analysis reveals alternative splicing complexity and transcriptomic diversity in porcine placentas across altitudes. DNA res..

[bib0024] Liu Y., Niu K., Wang R., Liang X., Lin C., Wu X., Zhai Z. (2023). Taurochenodeoxycholic acid inhibits intestinal epithelial cell proliferation and induces apoptosis independent of the farnesoid X receptor. Food Funct..

[bib0023] Liu J., Shi L., Li Y., Chen L., Garrick D., Wang L., Zhao F. (2021). Estimates of genomic inbreeding and identification of candidate regions that differ between Chinese indigenous sheep breeds. J. Anim. Sci. Biotechnol..

[bib0025] Luo W., Luo C., Wang M., Guo L., Chen X., Li Z., Zheng M., Folaniyi B.S., Luo W., Shu D., Song L., Fang M., Zhang X., Qu H., Nie Q. (2020). Genome diversity of Chinese indigenous chicken and the selective signatures in Chinese gamecock chicken. Sci. Rep..

[bib0026] Lyu S., Arends D., Nassar M.K., Weigend A., Weigend S., Wang E., Brockmann G.A. (2023). High-density genotyping reveals candidate genomic regions for chicken body size in breeds of Asian origin. Poult. Sci..

[bib0027] Mai C., Wen C., Xu Z., Xu G., Chen S., Zheng J., Sun C., Yang N. (2021). Genetic basis of negative heterosis for growth traits in chickens revealed by genome-wide gene expression pattern analysis. J. Anim. Sci. Biotechnol..

[bib0028] Mao H., Yin Z., Wang M., Zhang W., Raza S.H.A., Althobaiti F., Qi L., Wang J. (2022). Expression of DGAT2 gene and its associations with intramuscular fat content and breast muscle Fiber characteristics in domestic pigeons (Columba livia). Front. Vet. Sci..

[bib57] Millward D.J. (2012). Identifying recommended dietary allowances for protein and amino acids: a critique of the 2007 WHO/FAO/UNU report. Br. J. Nutr..

[bib0029] Mohebbifar A., Sedgh-Gooya S., Torki M. (2025). Optimizing productive performance of laying hen and egg quality under cold stress: a dietary approach with crude protein, methionine and zinc. Vet. Med. Sci..

[bib0030] Patterson N., Moorjani P., Luo Y., Mallick S., Rohland N., Zhan Y., Genschoreck T., Webster T., Reich D. (2012). Ancient admixture in human history. Genetics.

[bib0031] Peng X., Ed-Dra A., Song Y., Elbediwi M., Nambiar R.B., Zhou X., Yue M. (2022). Lacticaseibacillus rhamnosus alleviates intestinal inflammation and promotes microbiota-mediated protection against Salmonella fatal infections. Front. Immunol..

[bib0032] Purcell S., Neale B., Todd-Brown K., Thomas L., Ferreira M.A.R., Bender D., Maller J., Sklar P., de Bakker P.I.W., Daly M.J., Sham P.C. (2007). PLINK: a tool set for whole-genome association and population-based linkage analyses. Am. J. Hum. Genet..

[bib0033] Qiao H., Jiao B., Wang J., Yang Y., Yang F., Geng Z., Zhao G., Liu Y., Dong F., Wang Y., Zhou S. (2023). Comparative Analysis of miRNA Expression Profiles under Salt Stress in Wheat. Genes.

[bib0034] Rachman M.P., Bamidele O.., Dessie T., Smith J., Hanotte O., Gheyas A.A. (2024). Genomic analysis of Nigerian indigenous chickens reveals their genetic diversity and adaptation to heat-stress. Sci. Rep..

[bib0035] Ren X., Guan Z., Li H., Wen J., Zhao X., Wang G., Zhang X., Wang H., Zhang L., Yu F., Qu L. (2023). Extensive intra- and inter-genetic admixture of Chinese gamecock and other indigenous chicken breeds revealed by genomic data. Poult. Sci..

[bib0036] Sarkar C., Sadhukhan T., Bagh M.B., Appu A.P., Chandra G., Mondal A., Saha A., Mukherjee A.B. (2020). Cln1-mutations suppress Rab7-RILP interaction and impair autophagy contributing to neuropathology in a mouse model of infantile neuronal ceroid lipofuscinosis. J. Inherit. Metab. Dis..

[bib0037] Schmid M.J., Smith D.W., Burt B.L., Aken P.B., Antin A.L., Archibald C., Ashwell P.J., Blackshear C., Boschiero C.T., Brown S.C., Burgess H.H., Cheng W., Chow D.J., Coble A., Cooksey R.P.M.A., Crooijmans J., Damas R.V.N., Davis D., de Koning M.E, Delany T., Derrien T.T., Desta I.C., Dunn M., Dunn H., Ellegren L., Eory I., Erb M., Farre M., Fasold D., Fleming P., Flicek K.E., Fowler L., Fresard D.P., Froman V., Garceau P.P., Gardner A.A., Gheyas D.K., Griffin M.A.M., Groenen T., Haaf O., Hanotte A., Hart J., Hasler S.B., Hedges J., Hertel K., Howe A., Hubbard D.A., Hume P., Kaiser D., Kedra S.J., Kemp C., Klopp K.E., Kniel R., Kuo S., Lagarrigue S.J., Lamont D.M., Larkin R.A., Lawal S.M., Markland F., McCarthy H.A., McCormack M.C., McPherson A., Motegi S.A., Muljo A., Munsterberg R., Nag I., Nanda M., Neuberger A., Nitsche C., Notredame H., Noyes R., O'Connor E.A., O'Hare A.J., Oler S.C., Ommeh H., Pais M., Persia F., Pitel L., Preeyanon P., Prieto Barja E.M., Pritchett D.D., Rhoads C.M., Robinson M.N., Romanov M., Rothschild P., Roux C.J., Schmidt A., Schneider M.G., Schwartz S.M., Searle M.A., Skinner C.A., Smith P.F., Stadler T.E., Steeves C., Steinlein L., Sun M., Takata I., Ulitsky Q., Wang Y., Wang W.C., Warren J.M.D., Wood D., Wragg H., Zhou (2015). Third report on chicken genes and chromosomes 2015. Cytogenet. Genome Res..

[bib0038] Shen J., Hanif Q., Cao Y., Yu Y., Lei C., Zhang G., Zhao Y. (2020). Whole genome scan and selection signatures for climate adaption in Yanbian cattle. Front. Genet..

[bib0039] Shi J., Jiang S., Wang Q., Dong J., Zhu H., Wang P., Meng S., Zhang Z., Chang L., Wang G., Xu X., Xu P., Zhang Y. (2023). Spleen-based proteogenomics reveals that Escherichia coli infection induces activation of phagosome maturation pathway in chicken. Virulence.

[bib0040] Su C., Zhang L., Pan Y., Jiao J., Luo P., Chang X., Zhang H., Si X., Chen W., Huang Y. (2024). Enhancing aggression in Henan gamecocks via augmentation of serotonergic-dopaminergic signaling and attenuation of neuroimmune response. Poult. Sci..

[bib0041] Tran T.S., Kolodkin A..L., Bharadwaj R. (2007). Semaphorin regulation of cellular morphology. Annu. Rev. Cell Dev. Biol..

[bib0042] Vohra M., Kumar S., Sohnen P., Kaur S., Swamynathan S., Hirose T., Kozmik Z., Swamynathan S.K. (2025). Pard3 promotes corneal epithelial stratification and homeostasis by regulating apical-basal polarity, cytoskeletal organization and tight junction-mediated barrier function. Ocul. Surf..

[bib0043] Wan X., Xie B., Sun H., Gu W., Wang C., Deng Q., Zhou S. (2022). Exosomes derived from M2 type tumor-associated macrophages promote osimertinib resistance in non-small cell lung cancer through MSTRG.292666.16-miR-6836-5p-MAPK8IP3 axis. Cancer Cell Int..

[bib0044] Wang J., Liao J., Zhang F., Song D., Lu M., Liu J., Wei Q., Tang S., Liu H., Fan J., Zou Y., Guo D., Huang J., Liu F., Ma C., Hu X., Li L., Qu X., Chen L., Weng Y., Lee M.J., He T., Reid R.R., Zhang J. (2017). NEL-like molecule-1 (Nell1) is regulated by bone morphogenetic protein 9 (BMP9) and potentiates BMP9-induced osteogenic differentiation at the expense of adipogenesis in mesenchymal stem cells. Cell Physiol. Biochem..

[bib0045] Xiang H., Gao J., Yu B., Zhou H., Cai D., Zhang Y., Chen X., Wang X., Hofreiter M., Zhao X. (2014). Early Holocene chicken domestication in northern China. Proc. Natl. Acad. Sci. U. S. A..

[bib0046] Xing L., Li H., Miao D., Wei H., Zhang S., Xue Q., Wang H., Li J. (2024). Intermittent and mild cold stimulation enhances immune function of broilers via co-regulation of CIRP and TRPM8 on NF-kappaB and MAPK signaling pathways. Poult. Sci..

[bib0047] Xu L., Zheng L., Wang Z., Li C., Li S., Xia X., Zhang P., Li L., Zhang L. (2018). TNF-alpha-induced SOX5 upregulation is involved in the osteogenic differentiation of Human bone marrow mesenchymal stem cells through KLF4 signal pathway. Mol. Cells.

[bib0048] Xu L., He W., Tai S., Huang X., Qin M., Liao X., Jing Y., Yang J., Fang X., Shi J., Jin N. (2025). VCF2Dis: an ultra-fast and efficient tool to calculate pairwise genetic distance and construct population phylogeny from VCF files. Gigascience.

[bib0049] Xu N., Zhang L., Chen F., Feng Z., Zheng J., Li D., Zhao Y., Kang X. (2025). Population structure, selection signal and introgression of gamecocks revealed by whole genome sequencing. J. Anim. Sci. Biotechnol..

[bib0050] Xue Q., Zhang G., Li T., Ling J., Zhang X., Wang J. (2017). Transcriptomic profile of leg muscle during early growth in chicken. PLoS One.

[bib0051] Yang Y., Guan W., Sheng X., Gu H. (2024). Role of Semaphorin 3A in common psychiatric illnesses such as schizophrenia, depression, and anxiety. Biochem. Pharmacol..

[bib0052] Yang Z., Xu W., Liu Y., Sun T., Xiao C., Zou L., Zeng L., Deng J., Yang X. (2025). New insights into genetic architecture of Guangxi indigenous chickens using whole-genome sequencing. Poult. Sci..

[bib0053] Yin X., Fang W., Yuan M., Sun H., Wang J. (2023). Transcriptome analysis of leg muscles and the effects of ALOX5 on proliferation and differentiation of myoblasts in Haiyang yellow chickens. Genes (Basel).

[bib0054] Zhou H.J., Kong L..L., Zhu L.X., Hu X.Y., Busye J., Song Z.G. (2021). Effects of cold stress on growth performance, serum biochemistry, intestinal barrier molecules, and adenosine monophosphate-activated protein kinase in broilers. Animal.

