# Supplementary material for: Genetic architecture of two novel chicken breeds from Xinjiang: A whole-genome sequencing study on Ili gamecock and Yemili Chicken
Source: Poult Sci. 2026 Mar 21;105(7):106845. doi: 10.1016/j.psj.2026.106845 (PMC13087779; doi:10.1016/j.psj.2026.106845)
Supplement: Supplementary file 1 [file mmc1.docx]

**Overview List of Supplementary Figures**


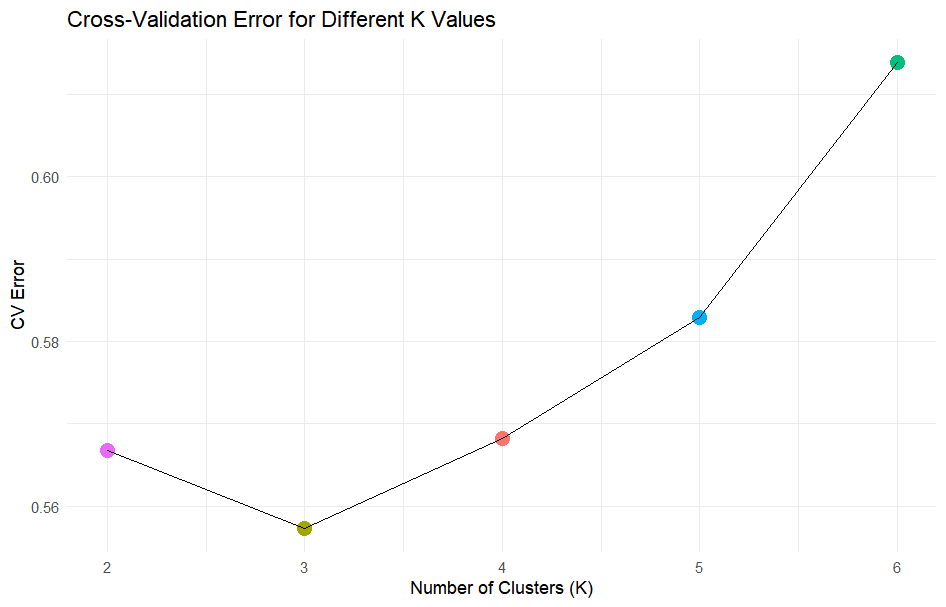


Figure. S1 Cross-validation (CV) error for varying values of K in the ADMIXTURE analysis.


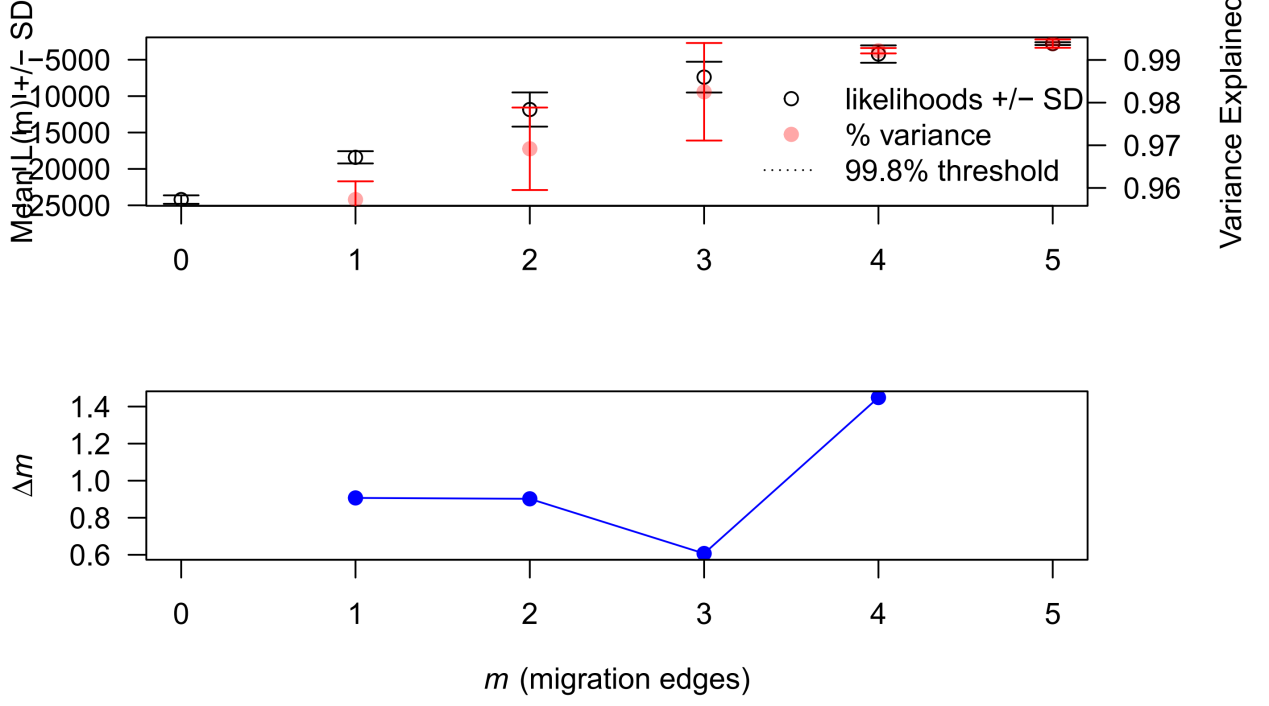


Figure. S2 Result OptM model fitting. The result of OptM evaluated different m results of TreeMix, demonstrating the best fit of TreeMix for m = 3 with the largest ∆m.


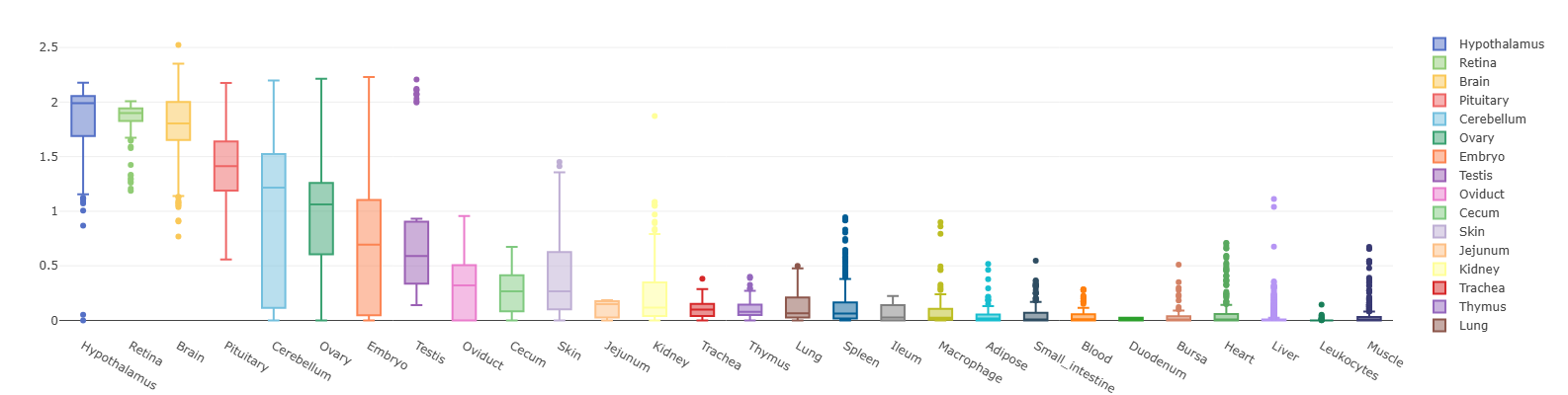


Figure. S3 *NELL1’s* Bulk tissue gene expression


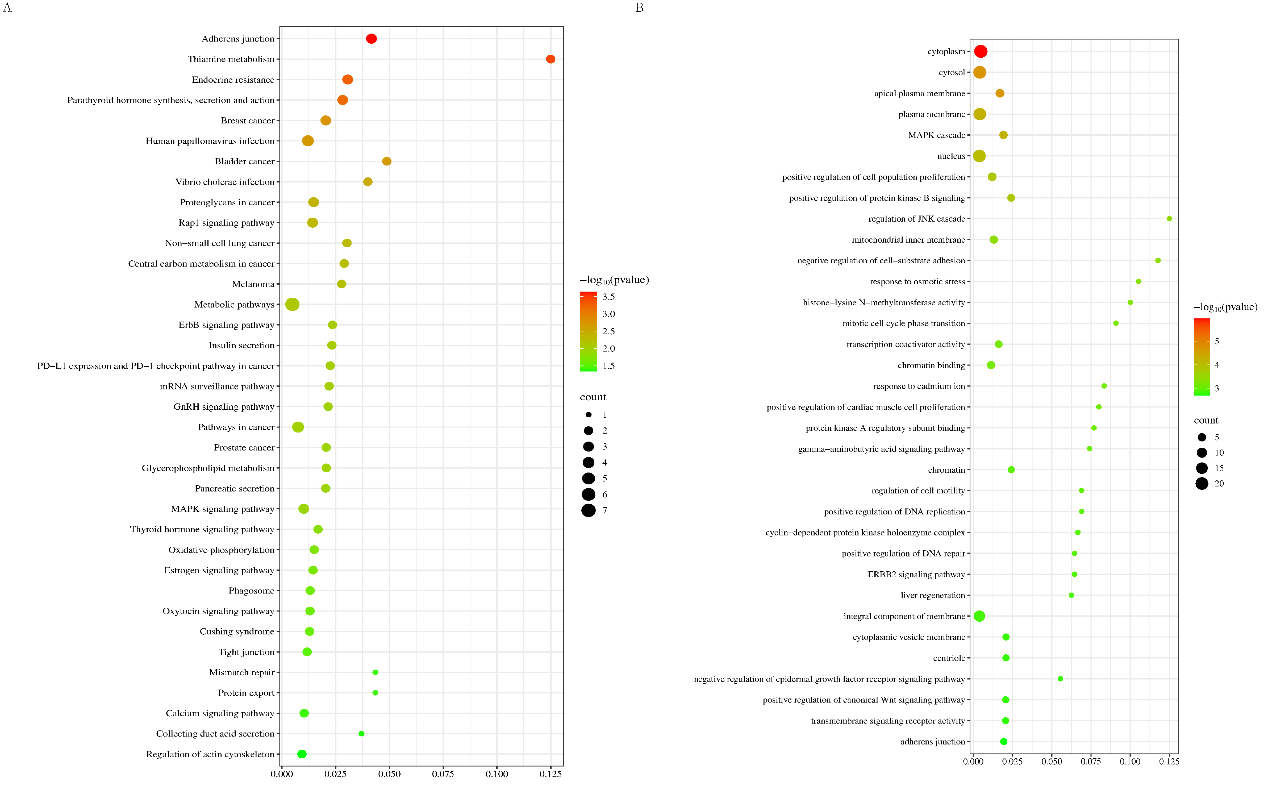


Figure. S4 YML’s Enrichment analysis:（A）Kyoto encyclopedia of genes and genomes;（B）Gene Ontology

**Supplemental files**

Additional file 1 Table S1

Format: .xlsx

Title: Supplementary Table S1. Sampling location and data statistics of different breeds

Description: Sample ID, Population (Abbrev), Origin.

Additional file 2 Table S2

Format: xlsx

Title: Supplementary Table S2. Quality information of sequencing data in this study

Description: Quality information of sequencing data

Additional file 3 Table S3

Format: xlsx

Title: Supplementary Table S3. Genetic diversity of all chicken breeds

Description: All genetic diversity in this study

Additional file 4 Table S4

Format: xlsx

Title: Supplementary Table S4. Top 1% genomic windows showing selection signatures identified by FST (Gamecock)

Description: The top 1% of differentiation regions, gene name of FST(Gamecock)

Additional file 5 Table S5

Format: xlsx

Title: Supplementary Table S5. Top 1% genomic windows showing selection signatures identified by π ratio (Gamecock)

Description: The top 1% of differentiation regions, gene name of π ratio (Gamecock)

Additional file 6 Table S6

Format: xlsx

Title: Supplementary Table S6. Overlapping candidate regions of top 1%global FST and π ration for the Gamecock

Description: Overlapping candidate regions for the Gamecock

Additional file 7 Table S7

Format: xlsx

Title: Supplementary Table S7. Top 1% genomic windows showing selection signatures identified by FST(YML)

Description: The top 1% of differentiation regions, gene name (FST)

Additional file 8 Table S8

Format: xlsx

Title: Supplementary Table S8. Top 1% genomic windows showing selection signatures identified by π ratio (YML)

Description: The top 1% of differentiation regions, gene name (π ratio)

Additional file 9 Table S9

Format: xlsx

Title: Supplementary Table S9. Overlapping candidate regions of top 1% global FST and π ration for YML chicken

Description: Overlapping candidate regions for YML chicken

Additional file 10Table S10

Format: xlsx

Title: Supplementary Table S10. Total GO terms and KEGG pathway of genes in Gamecock

Description: The results of GO and KEGG enrichment analysis in Gamecock

Additional file 11 Table S11

Format: xlsx

Title: Supplementary Table S11. Total GO terms and KEGG pathway of genes in YML

Description: The results of GO and KEGG enrichment analysis in YML

**Supplementary Figures**

Additional file 1 Figure S1

Format: doc

Title: Figure S1

Description: Cross-validation (CV) error for varying values of K in the ADMIXTURE analysis

Additional file 2 Figure S2

Format: doc

Title: Figure S2

Description: Result OptM model fitting. The result of OptM evaluated different m results of TreeMix, demonstrating the best fit of TreeMix for m = 3 with the largest ∆m

Additional file 3 Figure S3

Format: doc

Title: Figure S3

Description: *NELL1’s* Bulk tissue gene expression

Additional file 4 Figure S4

Format: doc

Title: Figure S4

Description: YML’s Enrichment analysis
